# Varying the rheological behavior of a micellar solution *via* modified microscopic structures in the presence of graphene oxide†

**DOI:** 10.1039/d5ra00366k

**Published:** 2025-03-25

**Authors:** Takumi Kusano, Norihiro Oyama, Hiroaki Yoshida, Hiroya Tanaka

**Affiliations:** a Toyota Central R&D Labs., Inc. Nagakute Aichi 480-1192 Japan kusano@mosk.tytlabs.co.jp

## Abstract

We experimentally and numerically investigate the aggregation structure of cationic wormlike micelles in the presence of graphene oxide (GO), in connection with the change in the rheological properties of their aqueous dispersion. We first confirm that the macroscopic viscoelastic properties under oscillatory shear vary non-monotonically with the addition of GO flakes. We then carried out three distinct experiments—small-angle X-ray scattering (SAXS) measurements, time-domain nuclear magnetic resonance (TD-NMR) measurements, and molecular dynamics (MD) simulations—to elucidate the structural modifications likely responsible for the rheological changes. The results of the SAXS and TD-NMR measurements suggest that surfactant molecules preferentially remain in a worm-like microparticle form when bonded to the GO surface but not completely covering the GO surface. Moreover, using MD simulations, we confirmed that the attractive interaction between negatively charged functional groups on the GO surface and cationic surfactants indeed leads to adsorption. Together with the results of the rheology measurements, the SAXS, TD-NMR, and MD simulation results suggest that GO flakes tend to form three-dimensional aggregates bridged by the wormlike micelles. Our results can be utilized to control the rheological properties of micellar solutions and provide a new paradigm for designing microscopic structures of GO.

## Abstract 2

We experimentally and numerically investigate the aggregation structure of cationic wormlike micelles in the presence of graphene oxide (GO), in connection with the change in the rheological properties of their aqueous dispersion.

## Graphical abstract

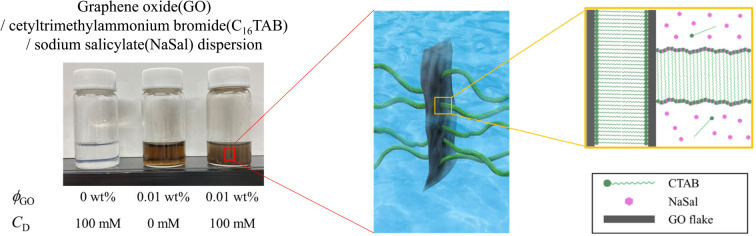

## 1 Introduction

Wormlike micelles, which are formed by surfactants in an aqueous electrolyte solution *via* self-assembled intermolecular aggregation,^1,2^ have attracted intensive attention over the past several decades. Various aspects such as thermodynamic, rheological, and structural properties have been investigated not only to achieve fundamental understanding^3–7^ but also to explore potential practical applications (*e.g.*, drag reduction agents^3,8^ and 3D printing gels^9^). One of the major features of micelles is their abundant structural degrees of freedom,^10,11^ which in turn leads to diverse rheological properties.^3,10,12^ In particular, Shikata *et al.*^13,14^ reported structural modifications of worm-like micelles (*e.g.*, scission and recombination) under steady and oscillatory shear. Such transitions of the micellar structure were shown to be closely related to the rheological property of solutions.^15–18^

Recently, several novel effects related to the electronic and chemical properties of two-dimensional materials, such as graphene and graphene oxide (GO), have been revealed.^19–22^ These properties are strongly dependent on the physicochemical structures of materials, such as their layering structure, and various techniques for controlling the structures have been explored. For example, several studies^9,23–26^ have found that interactions between GO and cationic molecules can modify the layering, structuring, and recovery of GO. Zou *et al.*^23^ used a charged polyelectrolyte to assemble three-dimensional structures composed of GO sheets. McCoy *et al.*^25^ used cationic surfactants to recover GO and Yue *et al.*^9^ applied GO/cationic wormlike micelle dispersions for 3D printing gels. In these systems, the cationic additives are commonly considered to be adsorbed onto GO, which modifies the interaction between the GO surfaces in the aqueous dispersion. Here, the cationic molecules promote the aggregation of GO flakes by destabilizing the dispersed state of them through surface charge neutralization. Importantly, such aggregation of GO flakes then modifies the structure of another major ingredient: micelles of cationic molecules, leading to a change in the macroscopic rheological property. In fact, a recent phenomenological study has shown that GO strongly influences the rheological behavior of cationic wormlike micelle solutions.^27^ However, studies on the microscopic structure of cationic worm-like micelles in the presence of these additives remain rare.^25,28^

In the present study, viscoelastic measurements as well as small-angle X-ray scattering (SAXS) measurements, time-domain nuclear magnetic resonance (TD-NMR) spectroscopy experiments, and molecular dynamics (MD) simulations were performed to investigate the effect of the addition of GO on the rheological and structural behaviors of wormlike micelles in a dispersion of cetyltrimethylammonium bromide (C_16_ TAB), sodium salicylate (NaSal) and GO.

## 2 Methods

### 2.1 Experimental

#### 2.1.1 Sample preparation

**Fig. 1:**
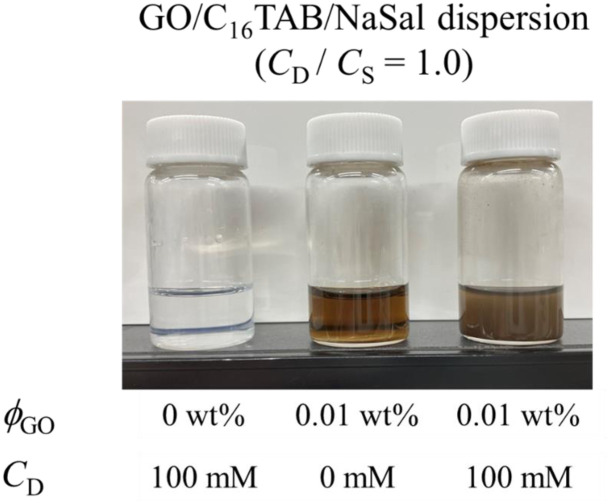
GO/C_16_TAB/NaSal dispersions at *C*_D_ = 100 mM. The molarity of C_16_TAB (*C*_D_) was set to be the same as that of NaSal (*C*_S_).

We prepared the GO/C_16_TAB/NaSal dispersions (Fig. 1) using the following procedures. C_16_TAB (Fujifilm Wako Pure Chemical) and NaSal (Fujifilm Wako Pure Chemical) were dissolved in water and a GO dispersion (Merck, 2 mg ml^−1^ dispersion in water) was added dropwise to the prepared C_16_TAB/NaSal solution. The resultant dispersion was agitated for 24 h using a magnetic stirrer at room temperature and sonicated at a frequency of 20 kHz for 1 h. Experiments were conducted using surfactant, additive salt, and GO dispersions in several different concentration combinations. Specifically, while the molarity of C_16_TAB, *C*_D_, and that of NaSal, *C*_S_, were kept constant and identical as *C*_D_ = *C*_S_ = 100 mM for all experiments (we provide some results with different value of *C*_D_ and *C*_S_ in the ESI†), results with various concentrations of GO (*ϕ*_GO_ = 0, 0.01, 0.05, and 0.1 wt%) were compared.

#### 2.1.2 Rheology

Rheological properties were measured using a rheometer (MCR302e, Anton Paar) with a cone plate radius of 50 mm and a cone angle of 1°. The frequency dependence of the storage modulus *G*′ and loss modulus *G*′′ were measured at strain *γ* = 1.0% and frequency *ω* in the range of between 0.1 and 100 rad s^−1^. The increment of the frequency at each step was chosen such that the interval was uniform on a base-10 logarithmic scale.

#### 2.1.3 Small angle X-ray scattering

SAXS measurements were performed at the BL8S3 beamline of the Aichi Synchrotron Radiation Center. The scattered X-rays were analyzed with an imaging-plate detector (R-AXIS IV++, Rigaku). The sample-to-detector distance was 1.1 m. The energy and wavelength of the scattered X-rays were 8.2 keV and 1.5 Å, respectively. The scattering vector *q* = (4π sin *θ*)/*λ* was varied in the range 0.02–0.4 Å^−1^, where 2*θ* is the scattering angle and *λ* is the wavelength. Notably, the measured range of the scattering vector enabled the measurement of lengths from 15 Å to 300 Å, which includes the length scale of C_16_TAB molecules (∼20 Å) (*i.e.*, the radius of the wormlike micelles). Because the scattering profiles were isotropic, the resultant two-dimensional SAXS images were converted into one-dimensional profiles by circular averaging. The corresponding background intensity of the capillary containing water was subtracted. For absolute intensity calibration, we used glassy carbon NIST Standard Reference Material.

#### 2.1.4 Time-domain nuclear magnetic resonance spectroscopy

NMR spectra were acquired using a MagnoMeter XRS spectrometer (Mageleka). We set the excitation carrier frequency to 12.4 MHz and the excitation-pulse width for 90° to 4.3 μs at 25 °C. We estimated the spin–spin relaxation time, *T*_2_, by analyzing the ^1^H NMR data using the Carr–Purcell–Meiboom–Gill method. This relaxation time, *T*_2_, becomes shorter when ^1^H atoms are adsorbed onto the carbon surface.^29^ To put it another way, this measurement, usually referred to as the TD-NMR measurement,^29,30^ enables a relative estimation of the amount of water molecules adsorbed onto GO surfaces.

### 2.2 Numerical simulations

We used MD simulations to investigate the interactions between the GO sheets and the surfactant molecules. We considered a system with an aqueous electrolyte solution between two parallel GO membranes. The model GO sheet was composed of pristine graphene sheets with hydroxyl groups, which were placed randomly on the basal plane such that the oxygen content ratio matched that of the commercial GO used in our experiments.^31^ The electrolyte solution contained the C_16_TAB surfactant molecules and the added salt NaBr. The concentrations of C_16_TAB and NaBr were both 100 mM. Here, we considered a different anion (Br^−^) for the added salt from the one employed in the experiments for numerical simplicity. The choice of the anion should play only a minor role and not affect the qualitative discussion on the interaction between GO substrates and surfactants here.

All the MD simulations were implemented using the open source code LAMMPS.^32^ The water molecules were modeled using the standard SPC/E model.^33^ The Lennard-Jones (LJ) parameters of AMBER96 were used for the carbon atoms of graphene, whereas the optimized potentials for liquid simulations all-atom (OPLS-AA) force field^34–36^ were used to model the hydroxyl groups, C_16_TAB molecules, and Na^+^ and Br^−^ ions. The partial charges assigned to atoms of C_16_TAB were taken from the work of Poorgholami-Bejarpasi and Sohrabi,^37^ who determined the partial charges using the restrained electrostatic potential (ResP) fitting approach. Throughout the simulations, each graphene sheet was kept rigid, and the hydroxyl group atoms were allowed to move freely. We used the SHAKE algorithm to maintain each water molecule as rigid.^38^ Long-range Coulomb interactions were treated by the particle–particle–particle–mesh (PPPM) method. Periodic boundary conditions were assumed in the transverse direction, and the non-periodicity in the longitudinal direction was dealt with by applying the periodic boundary condition with empty spaces outside the sheets. The artifacts from the image charges due to periodic conditions in the transverse direction were removed using the method of Yeh and Berkowitz.^39^ The velocity Verlet method was employed for the time integration of the Newton equation for each particle, with a time step of 1 fs. We maintained the temperature at 300 K using the Nose–Hoover thermostat (*i.e.*, canonical ensemble). The production runs were performed for 15 ns after equilibration for at least for 200 ps.

## 3 Results and discussion

This study aims to use four different measurements to investigate the changes in the rheological and structural properties of micellar solutions as a result of the addition of GO: viscoelastic rheological, SAXS, and TD-NMR experiments and MD simulations. In this section, the results of these measurements are presented.

### 3.1 Rheology (viscoelasticity)

**Fig. 2:**
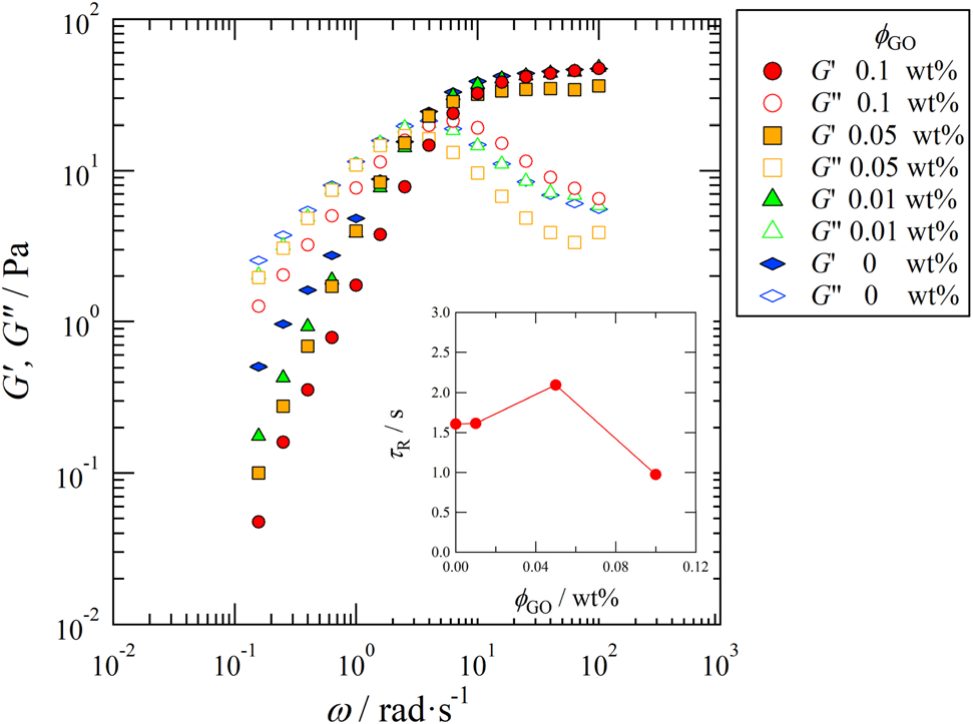
Log–log plot of the elastic modulus *G*′ and loss modulus *G*′′ as a function of the frequency *ω* at *C*_D_ = 100 mM. Symbols distinguish the concentration of the GO, *ϕ*_GO_, as indicated in the legend. (Inset) The stress relaxation time *τ*_R_ = 2π/*ω*_CO_ as a function of *ϕ*_GO_. The crossover frequency *ω*_CO_ is estimated by fitting the results to the single Maxwell model.

We first show in Fig. 2 the results of viscoelastic measurements of the GO/C_16_TAB/NaSal dispersions as a function of the frequency *ω*. The complex moduli *G*′ and *G*′′ presented here are well described by the single Maxwell model for all values of *ϕ*_GO_, which is consistent with the results of wormlike micelle solutions reported by Shikata *et al.*^13^ Fitting the results to the single Maxwell model allows us to estimate the stress relaxation time *τ*_R_ = 2π/*ω*_CO_, where *ω*_CO_ is the crossover frequency of the storage and loss moduli. The estimated value of *τ*_R_ varies non-monotonically with *ϕ*_GO_: it first increases with increasing *ϕ*_GO_ up to 0.05 wt%, then decreases when *ϕ*_GO_ further changes to 0.1 wt% (Inset of Fig. 2). Notably, this non-monotonic behavior contrasts the monotonic dependence of the viscosity on *ϕ*_GO_ (see Fig. S1 in ESI†). Since *τ*_R_ is proportional to the length of wormlike micelles^40–42^ (see eqn (3) appearing later), the results here also suggest that the length of the wormlike micelles changes non-monotonically with *ϕ*_GO_ as well. In the following subsection, SAXS measurements were performed to investigate the nanoscale structure of micelles in the presence of graphene oxide.

### 3.2 SAXS

**Fig. 3:**
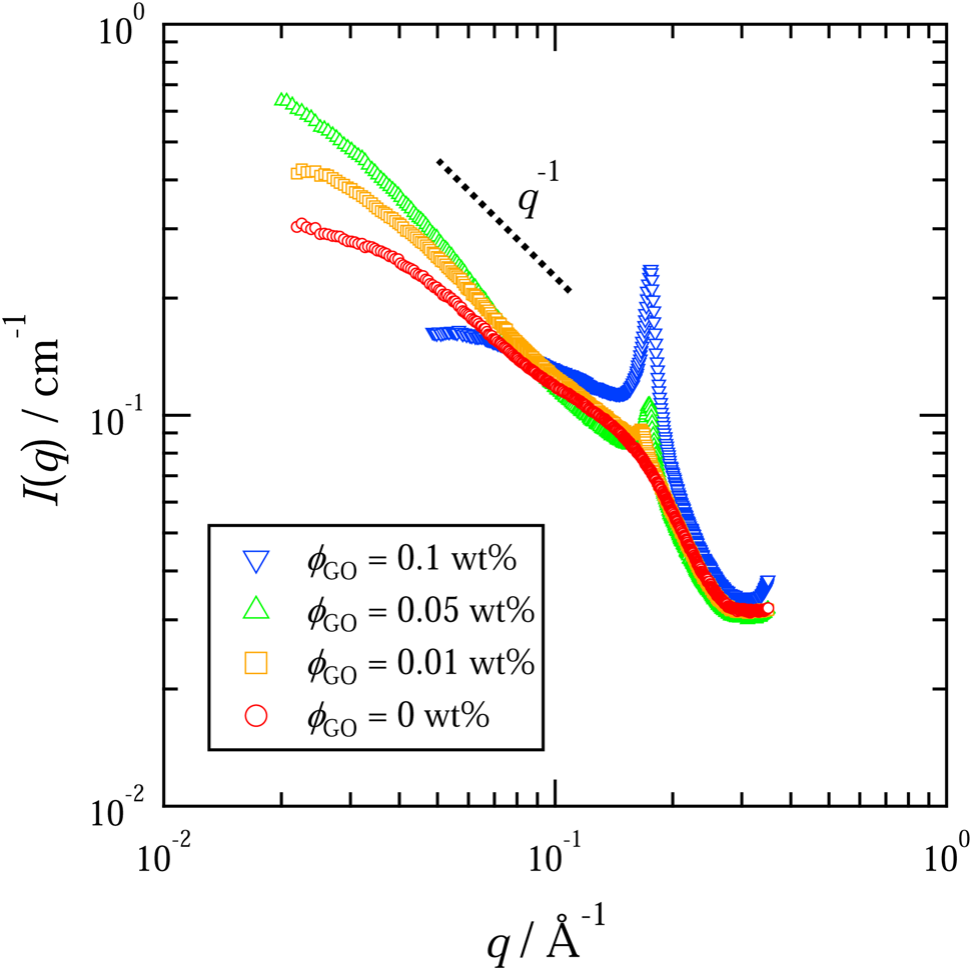
Scattering intensity *I*(*q*) as a function of the magnitude of the scattering vector *q*. Results for the system with *C*_D_ = 100 mM are shown. Different symbols represent different concentrations of GO, *ϕ*_GO_. The dashed line depicts the inverse proportional scaling relation *I*(*q*) ∼ *q*^−1^ that is typical behavior for rod-like particles.

Fig. 3 shows the scattering intensity *I*(*q*) as a function of the magnitude of the scattering vector *q*, which was obtained from SAXS measurements of the quiescent dispersion with *C*_D_ = 100 mM. Because the typical scaling behavior *I*(*q*) ∝ *q*^−1^ known for rod-like particles^10^ is observed for *ϕ*_GO_ ≤ 0.05 wt% in the low-*q* region (*q* < 0.07 Å^−1^), the wormlike micelles are likely to be formed in the GO/C_16_TAB/NaSal dispersion.

The scattering intensity exhibits another hallmark: we observe a peak at around *q* ≈ 0.17 Å^−1^ when GO is present. As reported in several articles^25,43^ and demonstrated by our MD simulations later, the GO flakes tend to adsorb the cationic surfactant molecules. This adsorption suppresses the electrostatic repulsion between GO flakes, enabling them to approach each other and allowing van der Waals interaction to become dominant, thereby greatly enhancing the aggregation. In fact, the positions of peaks in the SAXS profiles (Fig. 3), *q* ≈ 0.17 Å^−1^, are in accordance with the first Bragg peak attributed to the interval of the lamellar structure of the GO and C_16_TAB aggregates.^44^ In addition, the peak did not appear in the SAXS profile for the C_16_TAB/NaSal aqueous solution, and the peak intensity increased in proportion to *ϕ*_GO_. These results suggest that the peak is caused by the lamellar structure of GO, which is aggregated by the surfactants. This result is also consistent with the results of a previous study in which the adsorption of C_16_TAB onto GO was confirmed to promote the aggregation of GO sheets.^25^

### 3.3 TD-NMR

We next investigate the spin–spin relaxation time *T*_2_ using TD-NMR measurements. The value of *T*_2_ allows an indirect estimate of the amount of unbound water molecules in the system. In the present study, as explained below, we further utilize *T*_2_ to gain insight into the microscopic structures formed by GO and C_16_TAB.

**Fig. 4:**
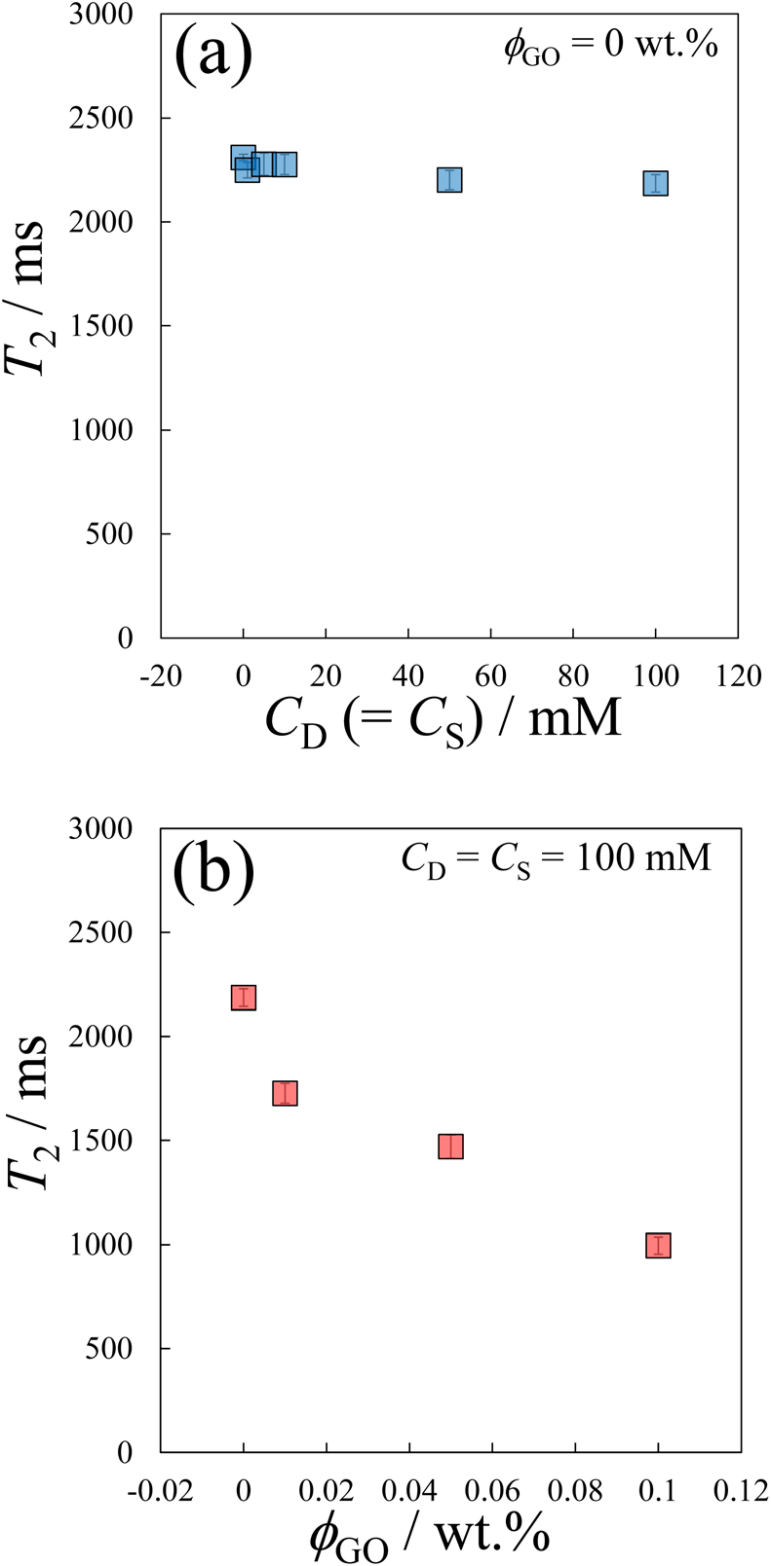
The relaxation time *T*_2_ as a function of (a) the concentration of surfactant, *C*_D_, and (b) the concentration of GO, *ϕ*_GO_. Shades depict the standard errors.

Fig. 4 shows the dependence of *T*_2_ on (a) *C*_D_ and (b) *ϕ*_GO_. In Fig. 4(a), *T*_2_ did not change significantly with respect to *C*_D_. On the other hand, in Fig. 4(b), *T*_2_ decreased monotonically as *ϕ*_GO_ increased. As shown in Fig. 4(a), the effect of micelle structure or dynamics on *T*_2_ is negligible compared with that of GO concentration. Therefore, the decrease in *T*_2_ with *ϕ*_GO_ in Fig. 4(b) is attributed to the interaction of water molecules and GO surface. This *T*_2_ decrease with *ϕ*_GO_ suggests that water molecules tend to be adsorbed on the GO surface. In other words, some adsorption sites on GO are occupied by water molecules even in the presence of C_16_TAB molecules. That is, the GO surfaces are not completely covered by these surfactants.

### 3.4 MD simulations

**Fig. 5:**
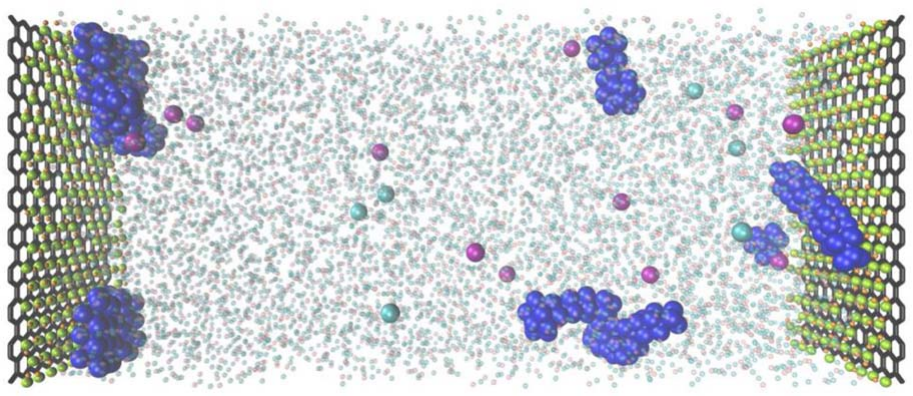
Snapshot of an MD simulation. Molecules are colored as C_16_TAB: blue, sodium ion: aqua, bromide ion: purple, hydroxyl groups: pink and yellow. Water molecules are illustrated transparently. The slab size is 24.595 Å × 42.6 Å × 100.64 Å, where the graphene is configured with 10 hexagonal lattices. The numbers of molecules are water: 3094, C_16_TAB: 6, and NaBr: 6.

**Fig. 6:**
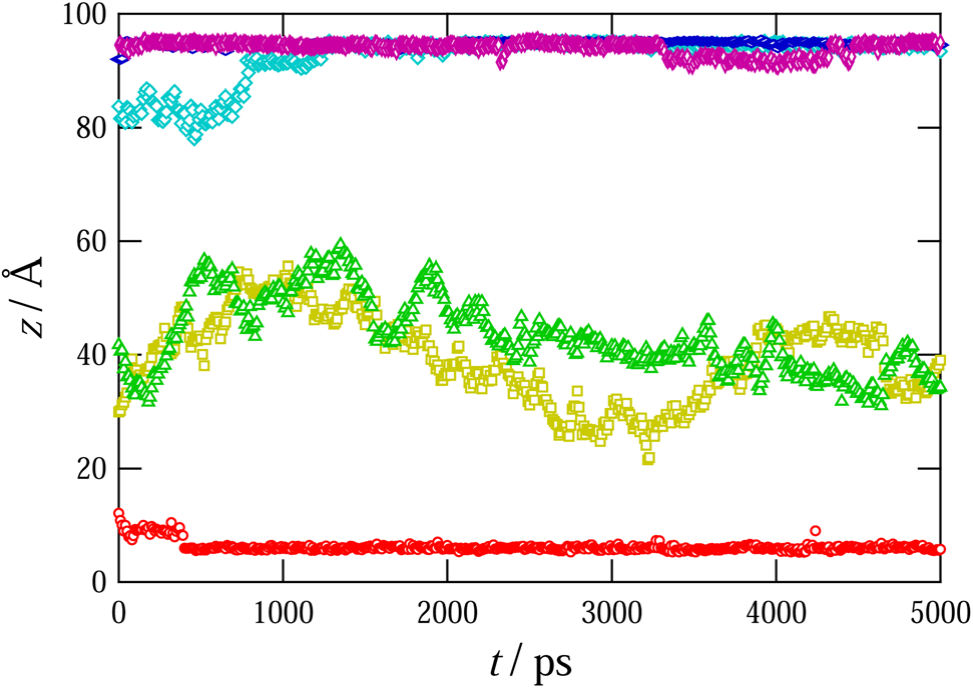
Time evolution of the positions of the N molecules in the surfactant molecules in the direction perpendicular to the GO membranes (*z*-direction). Different symbols are used to distinguish different molecules. The GO membranes are located at *z* = 0, 100.64 Å.

The GO flakes in water exhibit a strong negative surface potential due to the hydroxyl and carboxyl groups at their periphery. Although this resulting negative potential of GO flakes has been considered to attract the cationic surfactant molecules,^25,43^ we cannot directly assess the binding tendency experimentally. In the present study, we bypass this technical difficulty using MD simulations and numerically validate the attractive interactions between GO flakes and surfactants. Fig. 5 shows a snapshot of the system considered in our simulations. At the initial setup, C_16_TAB molecules were located around the center of the slab and were surrounded by water molecules (the number of C_16_TAB molecules is 6; refer to the caption of Fig. 5 for system details). As the simulation progressed, the C_16_TAB molecules moved in different random directions because of thermal fluctuations; some of the C_16_TAB molecules eventually reached the surface of the slab walls. The C_16_TAB molecules were trapped by the hydroxyl groups on the surface of the GO as a result of electrostatic attraction and the hydrophobic interaction. The trapping of C_16_TAB molecules is evident in the time evolution of the *z*-component (direction perpendicular to the GO membranes) of the nitrogen atoms of surfactants shown in Fig. 6. The figure shows that, indeed, once nitrogen atoms approach the GO surface, they cannot escape from it. This observation is strong numerical support for the attraction between GO and surfactant molecules in aqueous solutions.

### 3.5 Characteristic micellar lengths

**Fig. 7:**
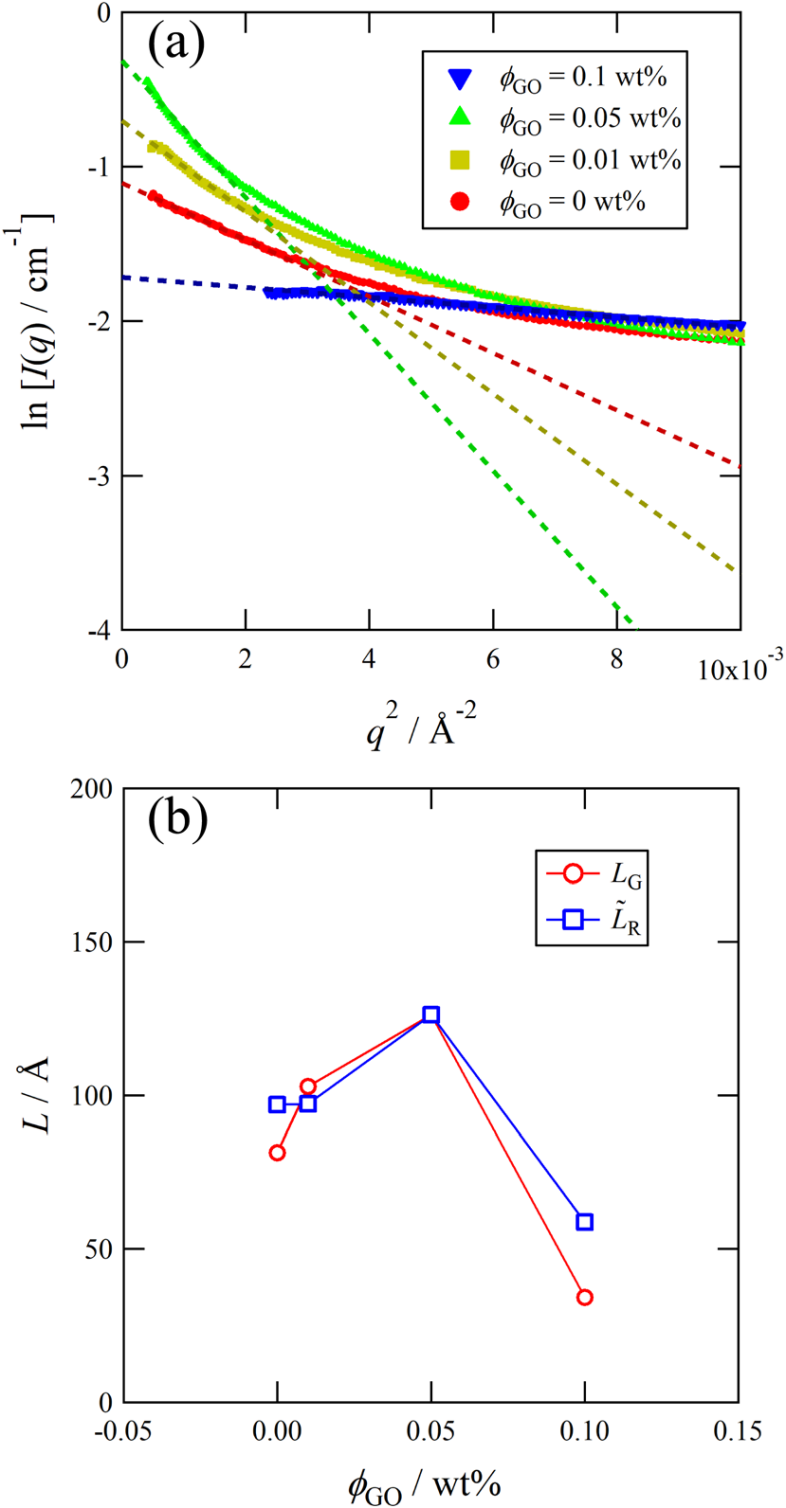
(a) Guinier plot of SAXS profiles with varying *ϕ*_GO_. The dashed line represents the fitted line of the Guinier model. (b) *ϕ*_GO_ dependence of *L*_G_ estimated from Guinier plot and *L̃*_R_ = *L*_R_ × (*L*^max^_G_/*L*^max^_R_) estimated from viscoelastic measurements.

The experimental and numerical measurements presented thus far provide us with two different ways to estimate the important microscopic structural information: the characteristic length scale of the wormlike micelles. Importantly, both these two distinct estimations consistently suggest non-monotonic dependence of the characteristic micelle lengths on the GO concentration *ϕ*_GO_. In this subsection, we explain the two estimation methods one by one. The first method relies on SAXS profiles. As mentioned above, the peak attributed to the lamellar structure of GO appeared in the SAXS profiles. Therefore, we performed Guinier analysis to separate the scattering profiles of the wormlike micelles from the peak profiles due to the lamellar structure. In Fig. 3, *I*(*q*) exhibits a shoulder (Guinier region) attributed to the scattering function of the wormlike micelles in the small-*q* range of 0.02 ≤ *q* ≤ 0.1 Å^−1^. A Guinier region provides an estimate of the gyration radius of scattering particles independent of particle shape. According to Guinier's approximation, the scattering function follows eqn (1) in the Guinier region.1
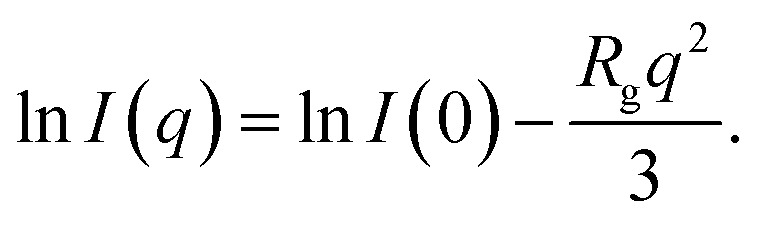
Therefore, the gyration radius of particles, *R*_g_, can be calculated from the slope of the Guinier plot.^45,46^Fig. 7(a) shows the Guinier plots based on the SAXS profiles in Fig. 3, with the slope of the fitted lines indicating the gyration radius *R*_g_ of the wormlike micelles. For a thin rod, the characteristic micellar length can be written using *R*_g_ as follows:^45,46^2
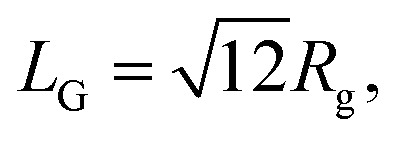
where we named the length estimated by this approach *L*_G_. In Fig. 7(b), the obtained value of *L*_G_ are plotted as a function of *ϕ*_GO_.

The length *L*_G_ changes in a non-monotonic fashion, with the maximum value located at *ϕ*_GO_ = 0.05 wt%.

The viscoelastic relaxation time *τ*_R_ of the wormlike micelles, which was reported in the inset of Fig. 2, can be utilized for the second estimation of the characteristic micellar length which we call *L*_R_ as:^40–42^3
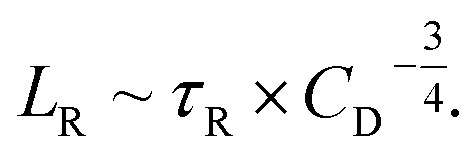
In Fig. 7(b), we compared *L*_R_ with *L*_G_. Notice that, since the precise value of *L*_R_ can be determined only up to an unknown proportional coefficient, we normalize it as *L̃*_R_ = *L*_R_ × (*L*^max^_G_/*L*^max^_R_), where *L*^max^_R_ and *L*^max^_G_ are the maximum values of two length scales and both of them are the values at *ϕ*_GO_ = 0.05 wt%. As can be seen in Fig. 7(b), *L*_G_ and *L*_R_ consistently indicate the non-monotonic dependence of micelle lengths on the GO concentration *ϕ*_GO_.

Now we discuss the cause of such a non-monotonicity of micellar lengths from the viewpoint of the interactions among GO, C_16_TAB, and NaSal. The results of our MD simulations showed that some surfactant molecules are adsorbed onto GO surfaces at an unassociated state, nor forming micellar structures (we call them “the adsorbed molecules” hereafter). The adsorbed molecules in the unassociated state here means that surfactant molecules that are adsorbed on GO but are not involved in the formation of the wormlike micelles. Since the adsorbed molecules form a lamellar structure with GO without NaSal, the molar ratio of NaSal (salt) to C_16_TAB (surfactant) in the bulk changes as a function of *ϕ*_GO_. The amount of the adsorbed molecules should increase with the concentrations of GO (*ϕ*_GO_). And this adsorption results in an increase in the salt-to-surfactant ratio in the bulk. The resulting relative superiority of the salt concentration then leads to an elongation of micelle length, which explains the cause of the experimentally observed increase in micelle length for *ϕ*_GO_ ≤ 0.05 wt% in Fig. 7(b). If we further increase the GO concentration *ϕ*_GO_, another effect becomes dominant: the overall decrease in the surfactant concentration in the dispersion. The surfactant deficiency at high GO concentration would result in shortening effects of micelle length: this is exactly what we observe for the case with *ϕ*_GO_ = 0.1 wt%.

### 3.6 Micellar structure

**Fig. 8:**
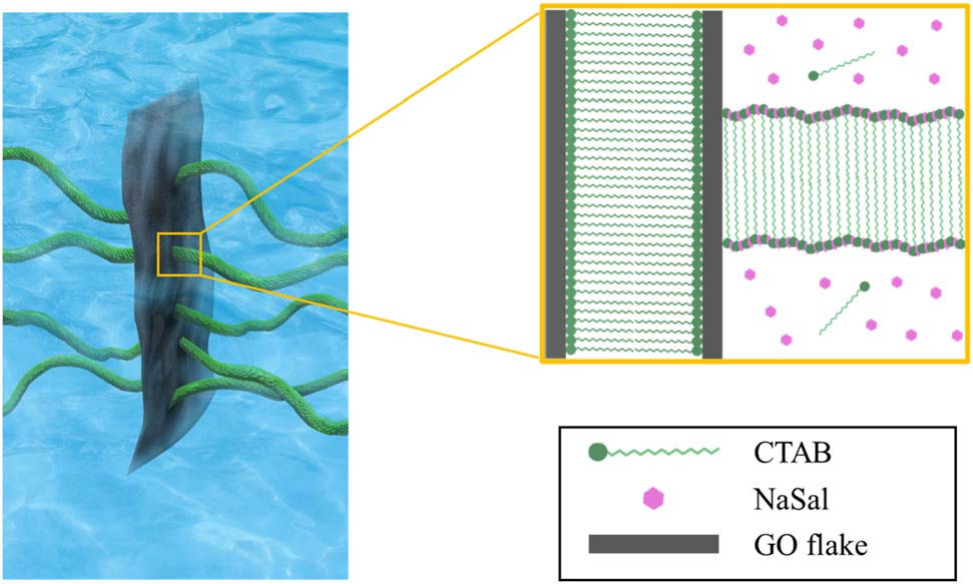
Schematic of the micellar structure in a GO/C_16_TAB/NaSal dispersion.

Finally, we provide a proposed sketch of the microscopic micellar structures in a GO/C_16_TAB/NaSal dispersion in Fig. 8. Our experiments provide three important implications for the microscopic structures.

First, as discussed in the previous subsection, both the micellar and the unassociated states of surfactant molecules coexist. Second, micelles and GO should self-organize into large aggregates that interfere with the visible light: the formation of large aggregate was evident in Fig. 1 where we visually showed that the GO/C_16_TAB/NaSal dispersion becomes turbid while pure C_16_TAB/NaSal solution and GO dispersion were transparent. Considering that the scattering intensity *I*(*q*) in Fig. 3 showed a clear peak at around *q* = 0.17 Å^−1^ corresponding to lamellar structure constituting of GO and C_16_TAB, we propose a stacking structure of surfactants-mediated bilayers of GO connected by the wormlike micelles as shown in Fig. 8. We stress that this structure is consistent with that reported previously.^27,28^ Finally, third, we emphasize that GO bilayers are not likely to be fully covered by micelles at least within the parameter space we investigated. This was conjectured based on the TD-NMR measurement results (Fig. 4) that indicated that water molecules occupy a fraction of adsorption sites.

## 4 Conclusion

We used rheological, SAXS, and TD-NMR measurements, in conjunction with MD simulations, to investigate the aggregation structure of cationic wormlike micelles and GO in aqueous dispersions. In viscoelastic measurements, the crossover frequency *ω*_CO_ of *G*′ and *G*′′ first decreases with increasing *ϕ*_GO_ up to *ϕ*_GO_ = 0.05 wt%, then starts increasing, indicating that the length of the wormlike micelles changes in a non-monotonic manner with *ϕ*_GO_. TD-NMR measurements were carried out to show that the surfactant molecules cover only part of the GO surface. Moreover, by performing MD simulations, we confirmed that the attractive interaction between negatively charged functional groups of the GO surface and cationic surfactants indeed leads to adsorption, as expected.^25,43^ Two micelle length estimated from SAXS profiles and viscoelastic relaxation time are both non-monotonically dependent on *ϕ*_GO_. We expect that some of the surfactant molecules are adsorbed onto the surface of GO in an unassociated state. The amount of these adsorbed surfactant molecules simply increases with increasing concentrations of GO (*ϕ*_GO_), which results in an increase in the salt-to-surfactant ratio in the dispersion. Because a higher salt concentration corresponds to a longer micelle length, the increase in salt concentration can facilitate micelle elongation. On the other hand, when *ϕ*_GO_ becomes high, large fraction of surfactant molecules are likely to be adsorbed onto the surface of GO flakes and the lack of surfactant molecules in the solution leads to the shortening of length of the wormlike micelles. Furthermore, together with the results of the rheology, the SAXS, TD-NMR and MD simulation results suggest that GO flakes tend to form a stacking structure of surfactant-mediated bilayers connected by the wormlike micelles. The present results provide a useful paradigm for controlling the rheological properties of micellar solution and designing the microstructure of GO membranes.

## Data availability

The data that support the findings of this study are available from the corresponding author on request.

## Conflicts of interest

There are no conflicts to declare.

## Supplementary Material

RA-015-D5RA00366K-s001
