# Supplementary material for: Varying the rheological behavior of a micellar solution via modified microscopic structures in the presence of graphene oxide
Source: RSC Adv. 2025 Mar 25;15(12):9034–40. doi: 10.1039/d5ra00366k (PMC11934230; doi:10.1039/d5ra00366k)
Supplement: RA-015-D5RA00366K-s001 [file RA-015-D5RA00366K-s001.pdf]

Supplementary Information (SI) for RSC Advances.
This journal is © The Royal Society of Chemistry 2025

Supporting Information : Varying the rheological behavior of a micellar solution via modified microscopic structures in the presence of graphene oxide

Takumi Kusano,* Norihiro Oyama, Hiroaki Yoshida, and Hiroya Tanaka

Toyota Central Research and Development Laboratories, Inc. Nagakute, Aichi, Japan

E-mail: kusano@mosk.tytlabs.co.jp

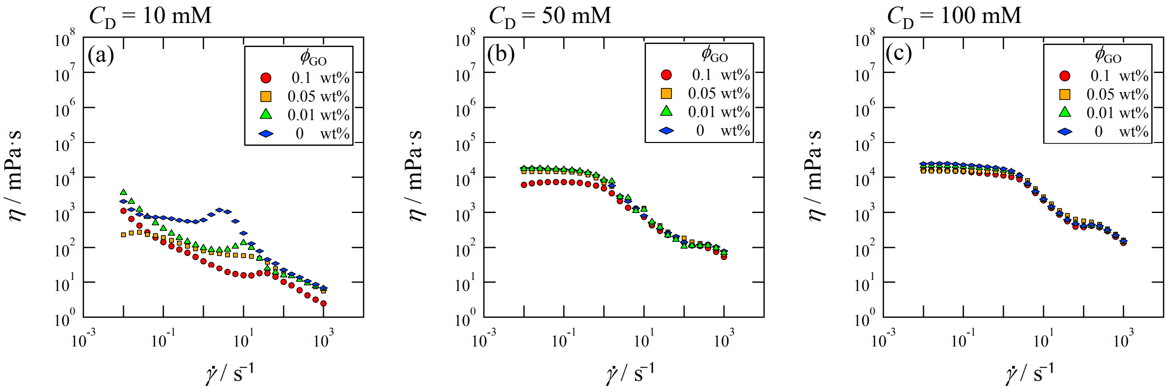

Figure S1: Log–log plot of the shear viscosity η of cetyltrimethylammonium bromide(CTAB)/sodium salicylate(NaSal)/graphene oxide(GO) dispersions as a function of the shear rate $\dot{\gamma}$. Different panels present results for systems with different concentrations of CTAB (C_{D}): (a) $C_{\mathrm{D}} = 10$ mM, (b) $C_{\mathrm{D}} = 50$ mM, and (c) $C_{\mathrm{D}} = 100$ mM. NaSal concentration is identical to CTAB concentration. Symbols distinguish the concentration of the GO, ϕ_{GO}, as indicated in the legend.
